# Antiproliferative Effects of Alkaloid Evodiamine and Its Derivatives

**DOI:** 10.3390/ijms19113403

**Published:** 2018-10-30

**Authors:** Xu Hu, Dahong Li, Chun Chu, Xu Li, Xianhua Wang, Ying Jia, Huiming Hua, Fanxing Xu

**Affiliations:** 1 Key Laboratory of Structure-Based Drug Design & Discovery, Ministry of Education, and School of Traditional Chinese Materia Medica, Shenyang Pharmaceutical University, Shenyang 110016, China; huxu105@163.com (X.H.); lidahong0203@163.com (D.L.); huimhua@163.com (H.H.); 2 School of Pharmacy, Shenyang Pharmaceutical University, Shenyang 110016, China; chuchunpharmacy@163.com; 3 Jiangsu Kanion Pharmaceutical Co., Ltd., Lianyungang 222001, China; lixu19900611@163.com; 4 School of Public Health, Qingdao University, Qingdao 266021, China; xianhuawang126@sina.com; 5 Faculty of Functional Food and Wine, Shenyang Pharmaceutical University, Shenyang 110016, China; jiayingsyphu@126.com; 6 Wuya College of Innovation, Shenyang Pharmaceutical University, Shenyang 110016, China

**Keywords:** evodiamine, alkaloid, antiproliferative effect, structural modification

## Abstract

Alkaloids, a category of natural products with ring structures and nitrogen atoms, include most U.S. Food and Drug Administration approved plant derived anti-cancer agents. Evodiamine is an alkaloid with attractive multitargeting antiproliferative activity. Its high content in the natural source ensures its adequate supply on the market and guarantees further medicinal study. To the best of our knowledge, there is no systematic review about the antiproliferative effects of evodiamine derivatives. Therefore, in this article the review of the antiproliferative activities of evodiamine will be updated. More importantly, the antiproliferative activities of structurally modified new analogues of evodiamine will be summarized for the first time.

## 1. Introduction

**Figure 1:**
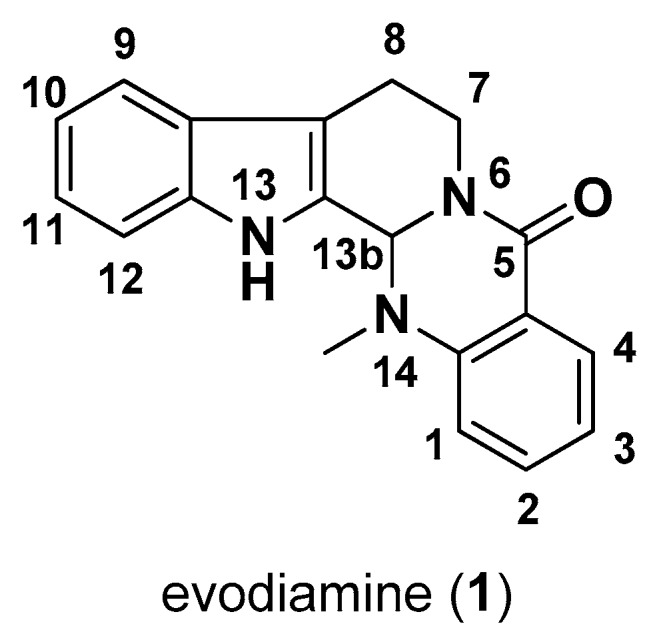
The structure and numbering of evodiamine (**1**).

Nature derived compounds are important for therapeutic leads [1,2,3,4,5,6,7]. Especially for anti-cancer agents, more than half cancer chemotherapy drugs are natural products and their derivatives [8,9,10]. Natural sources often provide privileged skeletons and define novel drug targets [11,12]. Among which, alkaloids, containing extra nitrogen atoms compared to other natural products, enrich structural diversity and some of them show better drug-like properties, such as drug-protein affinity and bioavailability. Most U.S. Food and Drug Administration approved plant derived anti-cancer agents are alkaloids, including vincristine, paclitaxel, and homoharringtonine. Evodiamine (**1**, Figure 1) is a famous alkaloid with a quinazolinocarboline skeleton. It was isolated from Evodia rutaecarpa, the dried and nearly ripe fruits of *Euodia rutaecarpa* (Juss.) Benth., *E. rutaecarpa* (Juss.) Benth var. *officinalis* (Dode) Huang, and *E. rutaecarpa* (Juss.) Benth var. *bodinieri* (Dode) Huang, three plants belonging to the family of Rutaceae. Evodiamine has been traditionally used to cure headache, amenorrhea, postpartum hemorrhage and gastrointestinal disorders. The biological activities of evodiamine have been widely investigated, including anti-obesity [13,14], anti-inflammatory [15,16], anti-atherosclerotic [17], neuroprotective [18,19], anti-gastrointestinal motility [20], and antiproliferative effects [21]. Among them, the antiproliferative activity of the multitargeting molecule evodiamine is attractive. 

In the past eight years, there have been five reviews on the biological activity of evodiamine. Evodiamine focusing on antiproliferative activities and molecular mechanisms have been reviewed by Jiang in 2009 [22], and Lu in 2012 [23]. In 2013, Yu et al. summarized the pharmacokinetics and the detailed exploration of target-binding properties of evodiamine [24]. Moreover, a patent review for therapeutic and cosmetic applications of evodiamine and its derivatives was reported by Gavaraskar in 2015 [25]. In 2016, Tan et al. reviewed the evodiamine functions and mechanisms of action in various chronic diseases [21]. To the best of our knowledge, there is no systematic review about the antiproliferative effects of evodiamine derivatives. This article updated the review of the antiproliferative activities of evodiamine. Moreover, the antiproliferative activities of structurally modified new analogues of evodiamine were summarized for the first time.

## 2. Pharmacological Activity

Previous studies evaluated the cytostatic effects of evodiamine in a variety of cancer cell lines. The cytotoxic effects of evodiamine in cancer cells were related to the induction of apoptosis, as well as inhibition of proliferation, migration, cell cycle progression, and angiogenesis. In addition, evodiamine was shown to regulate these cellular behaviors by affecting multi-targets. These works will be discussed in details.

### 2.1. Cytotoxic Effects in Lung Cancer Cells

Less than 15% of all lung cancers are small cell lung cancer (SCLC), and nearly 85% of lung cancer patients were diagnosed as non-small cell lung cancer (NSCLC) [26]. In human SCLC NCI-H1688 and NCI-H446 cells, G_2_/M arrest and the subsequent apoptosis were induced by evodiamine through mitochondria-dependent intrinsic apoptosis pathway rather than death receptor (DR)-induced extrinsic apoptotic pathway [27]. Moreover, evodiamine was shown to effectively increase the mitochondrial membrane depolarization and Bax/Bcl-2 ratio in human A549 NSCLC cell. The increased release of cytochrome c to cytosol activated intrinsic apoptotic pathway mediated by activation of caspase-9, -3, while increased release of cytochrome c to nuclear activated extrinsic apoptotic pathway mediated through activation of caspase-8. These studies indicated that evodiamine could induce apoptosis in NSCLC via both intrinsic and extrinsic pathways [28,29]. Further, the proliferation inhibition in A549 cells was induced by evodiamine, which was linked with the ability of evodiamine to increase the expression of oncoprotein metadherin, promote oxidative injury, arrest the cell cycle, and regulate the tumor-associated genes expression by controlling protein kinase B/ nuclear factor-κB (AKT/NF-κB) and sonic hedgehog/GLI family zinc finger 1 (SHH/GLI1) pathways [30,31]. Su et al. (2018) reported that evodiamine activate DNA methyltransferase 3A (DNMT3A)-induced neurogenic locus notch homolog protein 3 (NOTCH3) methylation, and subsequently induced growth inhibition in both A549 and H1299 NSCLC cells [32]. Moreover, the cytoprotective autophagy was induced by evodiamine in Lewis lung carcinoma (LLC) cells, and the combination of evodiamine with autophagy inhibitor therapy increased cell chemosensitivity [33]. Invasion activity of LLC cells in vitro and in vivo was shown to be decreased by evodiamine in previous studies [34,35]. As angiogenesis plays an important role in the development of lung carcinogenesis [36], the anti-angiogenesis activity of evodiamine was investigated by Shyu et al. (2006). Evodiamine was shown to decrease the expression of vascular endothelial growth factor (VEGF), a potent endothelial cell mitogen, in human lung adenocarcinoma CL1 cells. The conditioned medium derived from CL1 cells induced the formation of tube-like structures by human umbilical vein endothelial cells (HUVECs), while conditioned medium produced from evodiamine-pretreated CL1 cells exerted low ability of inducing capillary tube formation. Consistently, treatment of evodiamine could also decrease blood vessel density in chick embryo chorioallantoic membrane in vivo [37].

**Figure 2:**
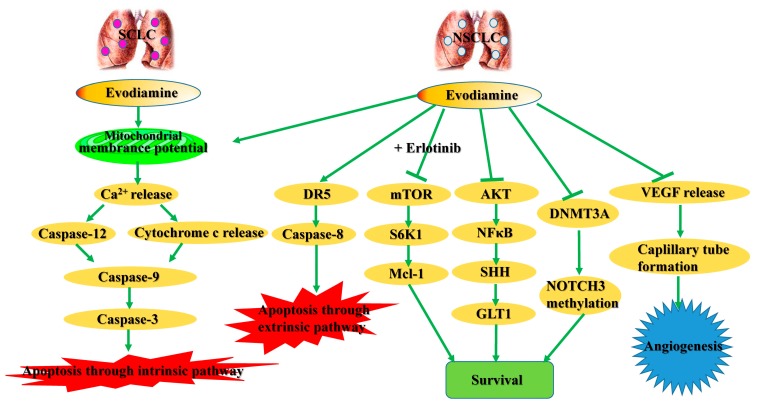
The schematic representation of cytotoxic effects of evodiamine.

Treatment of erlotinib is not effective in the treatment of NSCLC cells with wild-type epidermal growth factor receptor (EGFR) [38]. By inactivating mammalian target of rapamycin/p70 ribosomal S6 kinase 1/Mcl-1 (mTOR/S6K1/Mcl-1) pathway, evodiamine and erlotinib acted in synergy in inhibiting cell proliferation and increasing apoptosis in NSCLC cells (NCI-H460, NCI-H1299, NCI-H1650, and A549) with wild-type EGFR [39] (Figure 2).

The low water solubility of evodiamine limited its application [40]. To enhance the bioactivity of evodiamine in lung cancer cells, a previous study used the fructus bruceae oil-based emulsive nanosystems to increase the bioavailability of evodiamine in A549 cells [41]. Moreover, the antitumor effects of evodiamine were also enhanced with the emulsive nanosystems in A549-tumor-bearing mice [42].

### 2.2. Cytotoxic Effects in Liver Cancer Cells

Liver cancer ranks second among causes of cancer death, and about 90% of liver cancers worldwide are hepatocellular carcinoma (HCC) [43]. Yang et al. (2017) reported that evodiamine downregulated cell viability and inhibited cell cycle progression in human HCC HepG2 cells by decreasing p-Akt level and increasing the levels of apoptotic proteins Bax, cleaved-caspase-3 and cleaved-PARP (poly (ADP-ribose) polymerase) [44]. Signal transducer and activator of transcription signaling 3 (STAT3) is involved in the development of HCC, and the impact of STAT3 on evodiamine-induced cell death of HepG2 was determined by Yang et al. (2013). The study revealed that evodiamine suppressed the phosphorylation of Tyr^705^ of STAT3 leading to the growth inhibition of HepG2 cell line. Moreover, the expression of phosphatase shatterproof 1 (SHP-1) in HepG2 cells was increased by evodiamine treatment, and the inhibition of SHP-1 attenuated evodiamine-suppressed activation of STAT3 [45]. In another study, evodiamine induced the expression of tumor-suppressor WWdomain-containing oxidoreductase (WWOX) in human HepG2 and mouse Hepa1-6 cells. And the inhibition of WWOX reversed the cytotoxic effect of evodiamine. As such, the anti-hepatocellular carcinoma activity of evodiamine was linked with the activation of WWOX [46]. Activator protein 1 (AP-1) is required for tumor promotion [47]. Chao et al. (2011) reported that evodiamine effectively inhibited AP-1 activity and anchorage-independent transformation of human liver cells [48]. Furthermore, the progression of HCC is promoted under hypoxia stress, and a previous study evaluated the impact of evodiamine on growth of HCC cells (HepG2, Hep3B, and Huh-7) subjected to hypoxia. The cytotoxic effects of vorinostat, an anti-HCC drug, were shown to be increased by co-treatment with evodiamine through the downregulation of hypoxia-inducible factor 1α (HIF-1α) [49]. In addition, the combination of evodiamine with berberine acted in synergy in inducing cell death of SMMC-7721 human hepatocellular carcinoma cells [50].

Signaling pathways involved in angiogenesis are promising targets for HCC therapy, and evodiamine was shown to exert cytotoxic effects on HCCs (HepG2, SMMC-7721, H22) by downregulating angiogenesis, which was linked with inhibition of expressions of β-catenin and VEGFa [51]. Besides its role in regulating angiogenesis, the activation of β-catenin induced expressions of target genes that in turn promoted cell proliferation. The results indicated that β-catenin might be one of the factors that are critical to the development of HCC [52]. Yet, it remains unclear whether the downregulation of β-catenin expression contributes to evodiamin-induced cytotoxicity in HCC cells.

Qiu et al. (2016) incorporated evodiamine into hydroxypropyl-β-cyclodextrin (EVO/HP-β-CD) to increase its bioavailability in HCC cells. Compared with evodiamine alone, EVO/HP-β-CD exerted higher cytotoxic activities in human hepatoma HepG2 cells as evidenced by higher levels of apoptosis induced by the activation of caspase-3 [53].

### 2.3. Cytotoxic Effects in Leukemia Cells

**Figure 3:**
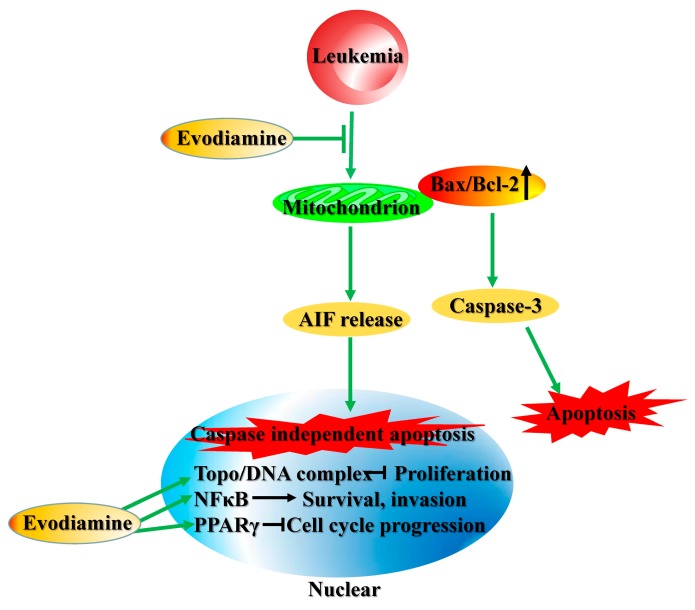
Cytotoxic effects of evodiamine in leukemia cells.

Leukemias, characterized by over-proliferation of abnormal blood cells, represent 31% of all cancers [54]. Evodiamine was shown to exert antiproliferative activity against human leukemia cells THP-1, K562, CCRF-CEM and camptothecin-resistant CCRF-CEM/C1 by inactivating topoisomerase I (topo I) and II (topo II). In addition, the formation of cleavage complexes of topoisomerases with DNA was not affect by evodiamine [55]. Evodiamine was also reported to cause the mitotic arrest and a consequent apoptosis in CCRF-CEM cells. And the study revealed that evodiamine-induced apoptosis was correlated with increased ratio of Bax/Bcl-2 and activity of caspase-3 [56]. However, benzyloxycarbonyl-Val-Ala-Aspfluoromethyl ketone (z-VAD-fmk), pan-caspase inhibitor, only partly attenuated the anti-apoptotic activity of evodiamine in human leukemic U937 cells, and overexpression of Bcl-2 failed to completely block the cytotoxic effect of evodiamine [57]. Further study showed that evodiamine treatment in U937 cells can also activate an apoptosis-inducing factor (AIF)-mediated apoptosis, which is not downregulated by Bcl-2 overexpression and caspase-independent. In addition, evodiamine is not toxic to human normal peripheral blood mononuclear cell (PBMC) [57,58]. In another study, peroxisome proliferator-activated receptor γ (PPARγ) signaling pathway was reported to be involved in the proliferation inhibition of evodiamine on K562 cells via inhibiting cyclin D1 and stimulating of p21, the important factors for the regulation of cell cycle progression [59]. Moreover, evodiamine decreased NF-κB activity and expressions of NF-κB-dependent antiapoptotic, proliferative, and metastatic genes in human myeloid leukemia KBM-5 cells, which indicated NF-κB might be another potential target of evodiamine for anti-leukemia therapy [60] (Figure 3).

### 2.4. Cytotoxic Effects in Breast Cancer Cells

Breast cancer accounts for 23% of all cancers, and is highly prevalent in females [61]. Evodiamine was reported to downregulate migration and upregulate apoptosis by inactivating phosphorylation of extracellular signal-regulated kinase (p-ERK) and activating p38 mitogen-activated protein kinase (MAPK) in human breast cancer MDA-MB-231 cells [62]. By arresting the cell cycle at G_0_/G_1_ phase and inducing apoptosis, evodiamine could also effectively reduce the viability of human breast cancer MCF-7 cells. Further, the combination of evodiamine with berberine possesses synergistic cytotoxic activity [63]. The regulation of expressions of ERα, ERβ, and PPARγ, as well as inactivation of Ras/MEK/ERK pathway, may also contribute to the growth inhibitory activity of evodiamine in breast cancer cell lines, such as T47D, MDA-MB-468, MCF-7, and MDA-MB-231 [64,65]. Moreover, evodiamine has been reported to inhibit topo I activity in MCF-7 cells by inducing stabilization of the complex of topo I and DNA, and subsequently inhibit the replication and transcription of DNA [66]. However, the data are controversial to the results published by Pan et al. (2012) showing that evodiamine interferes with the enzyme catalytic cycle rather than affecting the stabilization of the covalent complexes of enzyme-DNA [55]. Cancer stem-like cells (CSLCs) play an important role in tumor progression [67]. Han et al. (2016) enriched the CSLCs in the mammosphere-forming condition during culture of MDA-MB-231 or MCF-7 cells. Compared with bulk carcinoma cells, CSLCs were more sensitive to cytotoxic effects of evodiamine treatment. The selective activation of p53 and p21, and inactivation of Rb in CSLCs led to cell death at the G_1_/S checkpoint [68].

As the occurrence of multidrug resistance is a major problem for the therapy of breast cancer, the inhibitory activity of evodiamine against doxorubicin- and adriamycin-resistant human breast cancer NCI/ADRRES cells was determined in previous studies [69,70]. Evodiamine reversed phenomenon of apoptosis resistance through inhibition of expression of IAPs, upregulation of Bcl-2, and activation of Ras/MEK/ERK pathway, which subsequently disrupted the microtubule network and induced apoptosis of NCI/ADRRES cells.

Zou et al. (2016) developed a nanoparticulate delivery system based on PLGA nanoparticles (EVO-PLGA NPs) to enhance the uptake of evodiamine by breast cancer. Compared with evodiamine alone, the antiproliferation effects of EVO-PLGA NPs in MCF-7 cells were even more obvious. However, EVO-PLGA NPs failed to enhance the impact of evodiamine on G_2_/M cell cycle arrest, suggesting that the nanoparticles sustained evodiamine release [71].

### 2.5. Cytotoxic Effects in Ovarian Cancer Cells

Ovarian cancer is another common gynecological tumor, and >70% of patients ultimately die from the disease [72,73]. Chen et al. (2016) evaluate the cytotoxic activities of evodiamine in human epithelial ovarian cancer cell lines ES-2, SKOV-3, A2780, and A2780CP. Evodiamine induced apoptosis of these cancer cells via activation of double-stranded RNA-activated protein kinase-like ER kinase (PERK) and c-Jun N-terminal kinase (JNK), as well as disruption of mitochondrial membrane potential. The structure-activity relationships (SARs) study demonstrated that the alkyl group at position 14 of evodiamine plays an important role in inducing apoptosis in ovarian cancer cells [74]. Further, the suppression of PI3K/Akt pathway and downregulation of p38 MAPK and ERK1/2 expressions might also be correlated with evodiamine-induced cell death of human ovarian cancer cell line HO8910PM [75]. As chemoresistance is associated with poor prognosis in patients with ovarian cancer, Zhong et al. (2015) evaluated the impact of evodiamine on cell viability of chemoresistant epithelial ovarian cancer cells (paclitaxel-resistant A2780/PTX^R^ cells). Evodiamine was shown to enhance the chemosensitivity potential of paclitaxel-resistant A2780/PTX^R^ cells by downregulating MDR-1 expression, which indicated the potential of evodiamine in the treatment of chemoresistant ovarian cancer [76]. In addition, evodiamine resulted in G_2_/M arrest in HER-2/neu-overexpressing SKOV3 cells (taxol resistant ovarian cancer cell line), while the underlying mechanism is still unclear [77].

### 2.6. Cytotoxic Effects in Cervical Cancer Cells

The number of cervical cancer deaths is higher than any other gynecological tumor, and the development of cervical cancer is highly correlated with infections with carcinogenic human papilloma virus [78]. By arresting cell cycle at G_2_/M, evodiamine was shown to induce apoptosis in human cervical cancer HeLa cells with caspase-3, -8-dependent pathway [79]. However, a controversy study reported that evodiamine activated caspase-3 without affecting the activity of caspase-8, -1 in HeLa cells [80]. In addition, the increased ratios of Bax/Bcl-2 and Bax/Bcl-xL are responsible for apoptosis triggered by evodiamine-induced productions of reactive oxygen species (ROS) and nitric oxide (NO). Autophagy was shown to be activated by evodiamine at an early period and reduced at late stages, and the blockage of autophagy resulted in apoptosis in HeLa cells [81]. Further study also suggested that G_2_/M cell cycle arrest in HeLa cells was triggered by ROS/NO productions, which could be attenuated by the inhibition of protein tyrosine kinase (PTK) [82]. Moreover, DNA topo I was shown to be associated with poor prognosis in ovarian cancers, and evodiamine exerted inhibitory effect against topo I catalytic activity. Accordantly, evodiamine effectively decreased the tumor volume in the subcutaneous xenografts of the human ovarian cancer cell line, A2780^R2000^, in a mouse model [83].

### 2.7. Cytotoxic Effects in Glioma Cells

Glioblastomas is the most frequent type of gliomas with highly invasive behavior [84]. In human glioblastoma U87 cells, evodiamine was reported to increase the intracellular calcium level, and activate autophagy and apoptosis, while the induction of autophagy blocked the apoptosis. In another study, evodiamine increased cytosolic calcium level, autophagy and apoptosis in U87 cells, which was attenuated by the inhibition of endoplasmic reticulum (ER) calcium channel. The study indicated an important role of calcium in mediating autophagy and apoptosis pathways in U87 cells [85,86]. Consistently, Wu et al. (2017) found that evodiamine induced G_2_/M arrest and decreased the viability of both human glioblastoma U87 cells and rat glioblastoma C6 cells, while incubation with JNK inhibitor attenuated the effects of evodiamine [87]. In addition, the inactivation of PI3K/AKT pathway and upregulation of phosphorylation of p38 MAPK were also shown to be linked with the cytotoxic activity of evodiamine in human glioblastoma U251 and LN229 cells [88]. Moreover, evodiamine did not reduce cell viability of primary rat astrocytes. A study of SARs further showed that methyl at position 14 may be important for the cytotoxic effect of evodiamine against glioblastoma cells [87].

The tumor necrosis factor-α-related apoptosis-inducing ligand (TRAIL) could promote the apoptosis in cancer cells, while the resistance to TRAIL limits the effects of chemotherapy [89]. TRAIL alone failed to exert cytotoxic effect in human glioblastoma U87 cells. However, evodiamine increased the apoptosis promoted by TRAIL through the upregulation of expressions of DR4, DR5, and caspase-8, -3 [90].

### 2.8. Cytotoxic Effects in Colorectal Carcinoma Cells

Colorectal cancer is a common cancer in the elderly [91]. Evodiamine induced apoptosis of human colorectal carcinoma cells COLO-205 and HCT-116 via the upregulation of p53 and Bax/Bcl-2 ratio, as well as decreasing mitochondrial transmembrane potential [92]. In another study, evodiamine exerted a prominent effect on expression of DNMTs and microRNAs (miRNAs) during early tumorigenesis in rat malignant colonic tissue stimulated by transforming growth factor-β1 (TGF-β1). Among these evodiamine-regulated miRNAs, the increase of expression of miR-29a (targets DNMT3A/3B) was most obvious [93]. Liu et al. (2016) found that the level of miR-429 was upregulated in malignant human colorectal tissues compared with normal tissues, which was attenuated by evodiamine administration [94]. These studies indicated that different miRNA plays different roles in mediating anti-tumor effect of evodiamine. In addition, evodiamine exerted cytotoxic effect in human colon carcinoma HT-29 cells, which was attributed to the inhibition of Wnt/β-catenin pathway and telomerase activity [95,96,97]. Another study reported that the increased JNK activity also contributed to G_2_/M cell cycle arrest and apoptosis stimulated by evodiamine in human colon carcinoma cells COLO-205 and HT-29 [98]. Moreover, S-phase arrest, autophagy and caspase-dependent apoptosis were increased under the stimulation of evodiamine in human colon carcinoma Lovo cells, and the evodiamine-induced cell cycle arrest was attributed to the downregulation of the cyclin A and cyclin A-dependent kinase 2 (CDK2) expressions [99,100]. Further, the alkylation at position 14 was shown to play an important role in the cytotoxicity of evodiamine in colonic cancer cells [97], which is accordant with the previous study on ovarian cancer cells [74].

Previous studies showed that evodiamine decreased the invasion activity of colon 26-L5 cells in vitro and suppressed lung-specific metastasis of 26-L5 cells in mice in vivo [34,35,101]. The downregulated migration activity was revealed to be linked with the inactivation of Janus kinase/STAT3/matrix metalloproteinase 3 (JAK2/STAT3/MMP3) pathway in human colorectal cancer HCT-116 cells [102,103]. Moreover, the expression of midkine, an important growth factor for carcinogenesis, was inhibited by evodiamine in human colon cancer SW620 cells, which might be involved in the inhibitory activities of evodiamine against both invasion ability and anchorage independence growth of SW620 cells [104].

**Figure 4:**
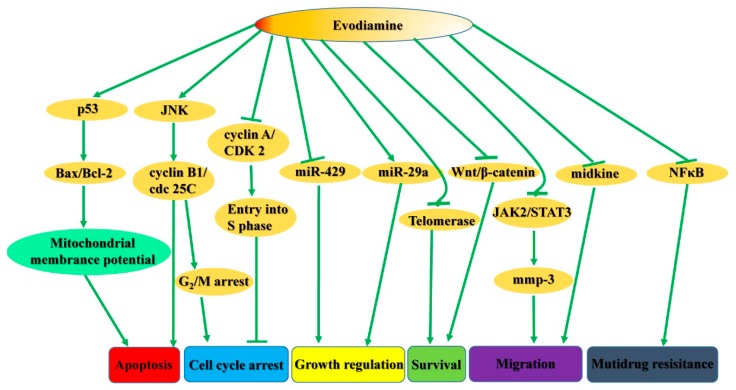
Sites of action of evodiamine against colorectal carcinoma cells.

Breast cancer resistance protein, also called ABCG2, is crucial for multidrug resistance (MDR) mechanism in colorectal carcinoma. Evodiamine was shown to suppress ABCG2-mediated drug resistance against oxaliplatin (L-OHP)-resistant HCT-116/L-OHP cells via inactivation of NF-κB in vitro. And the activation of NF-κB and colorectal tumor growth in a MDR xenograft model were inhibited in vivo [105] (Figure 4). In contrast, evodiamine was shown to upregulate NF-κB and p53 levels by down-regulating histone deacetylase 3 in HCT-116 xenografts in another study [106].

### 2.9. Cytotoxic Effects in Gastric Cancer Cells

Gastric cancer, a leading cause of cancer death, is frequently diagnosed at advanced stages [107]. Yue et al. (2013) revealed that evodiamine inhibited the viability of cells from gastric tumor tissues, which was correlated with downregulation of thymidylate synthase (TS) and excision repair cross-complementing 1 (ERCC1) [108]. Besides, evodiamine increased the beneficial effect of radiation treatment for human gastric carcinoma BGC-823 cells. The inactivation of Her-2/AKT/Bcl-2 pathway was further revealed to be correlated with the cytostatic activity of evodiamine [109]. In human gastric carcinoma SGC-7901 cells, evodiamine promoted proliferation and arrested the cell progression at G_1_ and G_2_/M phases, which further contributed to the cytotoxic and carcinogenic mechanisms of evodiamine [110,111,112,113,114]. Shen et al. (2015) reported that evodiamine also upregulated apoptosis through the upregulation of caspase-3 and survivin in SGC-7901 cells [115]. However, z-VAD-fmk only partially blocked evodiamin-promoted apoptosis in SGC-7901 cells [116]. The upregulation of acid sphingomyelinase (aSMase) and the downregulation of sphingomyelin were found to be the caspase-independent mechanism for evodiamine-induced apoptosis in SGC-7901 cells [117]. Moreover, evodiamine exerted inhibitory effect against the invasion activity of SGC-7901 cells through downregulation of midkine and polo-like kinase 1 (PLK1) expressions [118,119]. Gastric cancer stem cells were shown to promote tumorigenesis, metastasis and drug resistance [120]. Wen et al. (2015) reported that evodiamine could inactivate the Wnt/β-catenin pathway and subsequently suppress stem cell potential and proliferation of gastric cancer stem cells and repress the epithelial-to-mesenchymal transition [121]. However, evodiamine increased the secretion of IL-8, the cytokine promotes cell invasion, from human gastric cancer AGS cells, while berberine could counteract this side-effect of evodiamine. The study provides a safe strategy implementing combination therapy with evodiamine and berberine [122].

### 2.10. Cytotoxic Effects in Melanoma Cells

Melanoma is the primary skin cancer with ~132,000 cases worldwide annually [123]. The impact of evodiamine on invasion activity of melanoma was determined in previous studies using B16-F10 melanoma cells [34,35]. And these studies showed that the invasion potential of B16-F10 cells in transwell migration assay was effectively inhibited by evodiamine treatment.

In A375-S2 melanoma cells, the downregulation of protein kinase C (PKC) and silent information regulator of transcription (SIRT1), as well as the upregulation of Bax/Bcl-2 ratio, were correlated with evodiamine-induced increase of apoptosis. Evodiamine could also induce necrosis by activating p38 MAPK and inactivating ERK in A375-S2 cells [79,124,125,126,127]. During the course of evodiamine-induced apoptosis, the upregulated intracellular ROS followed by an onset of mitochondrial depolarization contributes to both Fas-mediated extrinsic and mitochondria-mediated intrinsic apoptosis pathways [128]. Evodiamine exerted a strong inductive effect on the production NO in A375-S2 cells, while the inactivation of p38 MAPK and NF-kB blocked evodiamine induced NO synthesis and cell damage [129]. Wang et al. (2005) found that the inhibition of interleukin 1 (IL-1) receptor in A375-S2 cells effectively attenuated the increased evodiamine-induced expressions of Fas-ligand (Fas-L), caspas-8, -3, and the phosphorylated p38 MAPK, which indicated IL-1-induced death cascade might be one of the targets for evodiamine [130]. Moreover, MG132, the inhibitor of ubiquitin-proteasome, further increased the sensitivity of A375-S2 cell to evodiamine induced cell death through the upregulation of the expressions of Fas-L, Bcl-2, and caspase-3 [131].

### 2.11. Cytotoxic Effects in Thyroid Cancer Cells

Thyroid carcinoma accounts for 1.3–1.5% of all cancers [132]. Lv et al. (2016) reported that evodiamine-treated human papillary thyroid cancer K1 cells underwent apoptosis that was correlated with inactivation of PI3K/Akt pathway [133]. Moreover, increased levels of G_2_/M arrest and caspase cascade-induced apoptosis contributed to evodiamin-induced cytotoxicity in human anaplastic thyroid cancer ARO cells [134]. Accordantly, the tumorigenesis of ARO in a xenograft mice model was inhibited by evodiamine in vivo [135]. The changes in protein expression patterns in ARO after evodiamine stimulation were further determined by Yu et al. (2018). And 77 proteins involved in transcriptional control, protein folding, and lipid metabolism contributed to evodiamine-induced cytotoxicity in ARO cells [136].

### 2.12. Cytotoxic Effects in Nasopharyngeal Carcinoma Cells

Nasopharyngeal carcinoma, the poorly understood cancer, arises from the nasopharynx epithelium [137]. Peng et al. (2015) reported an inhibitory role of evodiamine on the migration and invasion of human nasopharyngeal carcinoma HONE1 and CNE1 cells without affecting cell proliferation. Further investigation revealed that evodiamine downregulated cancer invasion- and metastasis-associated MMP-2 activity, which was controlled by NF-кB p65 nuclear translocation and the ERK1/2 phosphorylation [138].

### 2.13. Cytotoxic Effects in Renal Cancer Cells

Renal cell carcinoma usually develops from the lining of kidney tubules, and it accounts for ~90% of all renal cancer [139]. Evodiamine was shown to induce apoptosis in human renal cell carcinoma Caki-1, ACHN, A498, and 786-O cells, which was attenuated by stimulation with inhibitors of PERK or JNK. A methyl at position 14 was shown to be important for evodiamine’s cytotoxic action in renal carcinoma cells in a structure activity study [140], which is accordant with the previous study on glioblastoma cells [87]. Moreover, a transcriptome analysis using Caki-1 cells indicated that evodiamine-regulated genes in Caki-1 cells were mainly involved in apoptosis and cell cycle [141].

### 2.14. Cytotoxic Effects in Bladder Cancer Cells

Bladder cancer is a common genitourinary cancer with 70% recurrence rate [142,143]. It has been shown that human bladder cancer cells 253J and T24 underwent apoptosis in response to the stimulation of evodiamine. In addition, evodiamine enhanced TRAIL-induced apoptosis through the regulation of mTOR/S6K1/Mcl-1 pathway [144]. On the other hand, evodiamine arrested cell cycle progression in G_2_/M phase and increased caspase-dependent apoptosis in bladder tumor cell lines 5637 and HT1197, and the effects of evodiamine was linked with the activations of p38 MAPK and JNK [145].

### 2.15. Cytotoxic Effects in Pancreatic Cancer Cells

Pancreatic cancer is a leading cause of death from cancer with limited effective therapies [146]. Evodiamine was shown to induce apoptosis via inhibition of NF-κB activation, and subsequently upregulated Bax/Bcl-2 ratio and ultimately induced apoptosis in human pancreatic tumor cell PANC-1 [147]. Accordantly, evodiamine inactivated NF-κB in human pancreatic tumor cell SW1990, while gemcitabine was shown to induce the activation of NF-κB. These findings were consistent with a previous study researching the impact of evodiamine on activation of NF-κB in various carcinoma cell lines [60]. As the activation of NF-κB is one of the reasons for the development of chemoresistance during cancer therapy, the inactivation of NF-κB by evodaimine may sensitize pancreatic cancer cells to anti-tumor therapies [148].

### 2.16. Cytotoxic Effects in Prostate Cancer Cells

Prostate cancer is one of the leading causes of cancer-related death in males [149]. Through inhibition of microtubule spindle formation, evodiamine was shown to effectively arrest cell cycle progression in G_2_/M phase and subsequent induce apoptosis in human prostate cancer PC-3 and DU145 cells [150,151]. And the cytotoxic activities of evodiamine may attributed to the activation of cyclin B1/p34^cdc2^ complex and caspase-3, -9 [152].

### 2.17. Cytotoxic Effects in Osteosarcoma Cells

Osteosarcoma is the most common bone sarcoma in children and young adults [153]. Evodiamine was shown to inhibit the proliferation of human osteosarcoma 143B02 cells through inactivation of PTEN/PI3K/Akt pathway [154]. By inactivating Raf/MEK/ERK signaling pathway, evodiamine also induced growth inhibition and inactivated the activities of migration and invasion of U2OS cells [155,156]. Moreover, the inactivation of Wnt/β-catenin signaling pathway contributed to the cytotoxic activity of evodiamine in human osteosarcoma MG-63 cells [157].

### 2.18. Cytotoxic Effects in Oral Cancer Cells

Oral cancer is common in men in developing countries [158]. The apoptosis was induced in human oral cancer cells (MC3 and HSC4) by evodiamine though activation of caspase-3 and PARP cleavage. Further studies indicated the AKT/myeloid cell leukemia 1 (Mcl-1) signaling pathway is responsible for the observed cytotoxicty of evodiamine in oral cancer cells [159].

### 2.19. Cytotoxic Effects in Salivary Adenoid Cystic Carcinoma Cells

Salivary adenoid cystic carcinoma, the cancer with varied morphologic and clinical manifestations, is an uncommon salivary gland malignancy [160]. The effect of evodiamine on salivary adenoid cystic carcinoma SACC-M cell growth regulation was determined by Chen et al. (2012). And the study revealed that evodiamine significantly decreased SACC-M cell viability through downregulation of proliferation and upregulation of apoptosis [161].

## 3. Advances in Antiproliferative Evodiamine Derivatives

Naturally selected chemical scaffolds with bioactivity can be regarded as privileged structures. However, the probability for direct use of natural product as therapeutic drug is very low. The most common method is to generate analogues by structural modification approaches [162,163,164,165,166,167]. Although evodiamine has been well studied for its antiproliferative effects, it is still unqualified to be approved for clinical use. The physicochemical properties and antitumor potency remain to be significantly improved. For better design derivatives and understand the SARs, the existing medicinal chemistry work of evodiamine is summarized as follows.

**Scheme 1:**
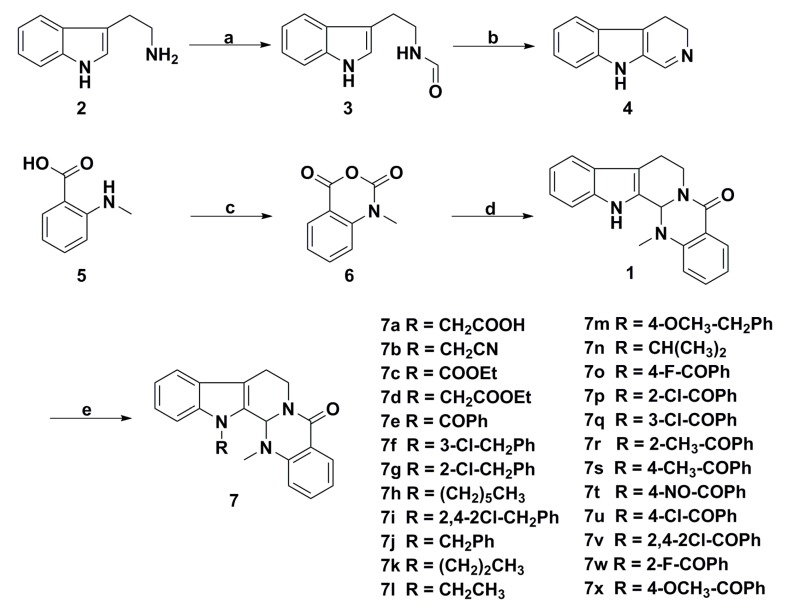
Synthetic route of evodiamine **1** and evodiamine derivatives **7a**–**x**. Reagents and conditions: (**a**) HCOOEt, reflux, 12 h; (**b**) POCl_3_, 0 °C 2 h, then rt, 2 h; (**c**) ClCOOEt, reflux, 12 h; (**d**) **4**, 30–60 °C, 6 h; (**e**) RX, NaH, 80 °C, 24 h.

In 2010, Dong et al. found evodiamine as human Topo I inhibitor by structure-based virtual screening and lead optimization [168]. Subsequently, by introducing alkyl, benzoyl and benzyl groups and ester at N-13, evodiamine derivatives were synthesized as Topo I inhibitors (Scheme 1). The results showed that the substituted benzoyl groups were favorable for the antiproliferative activity and spectrum. Among them, *N*-benzoyl analogue **7u** showed the strongest antiproliferative effects against A549, HCT-116 and MDA-MB-435 human cancer cell lines with IC_50_ values of 0.86, 2.6 and 0.049 μM, respectively. In addition, in vitro Topo I inhibition assay also demonstrated that **7u** was stronger than evodiamine and was active (at 100 μM) against the relaxation of supercoiled DNA, which was consistent with their docking results.

**Figure 5:**
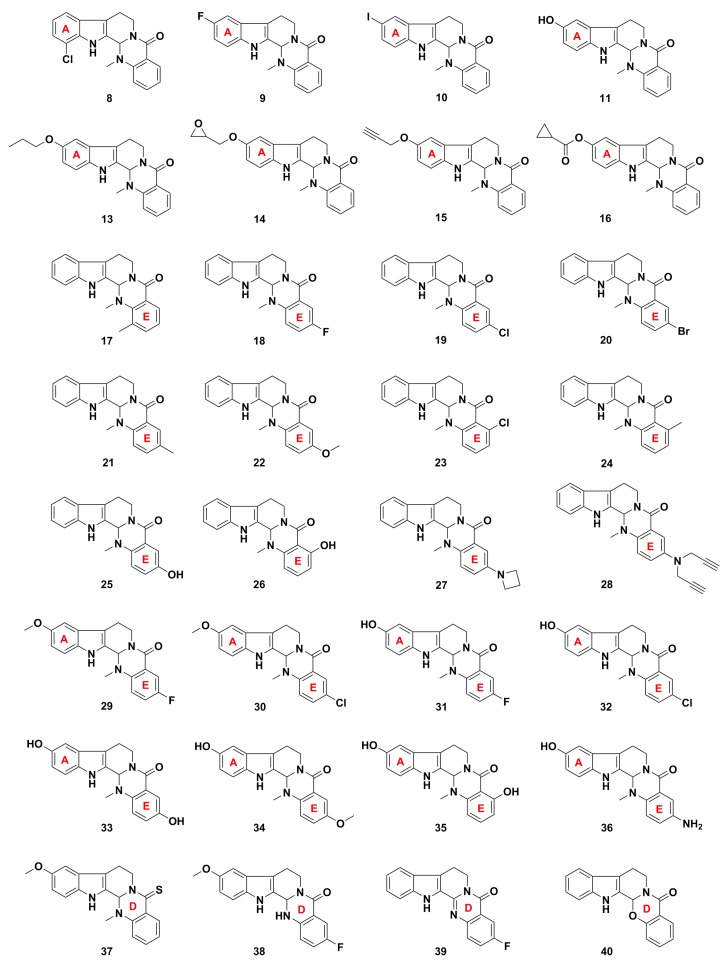
Chemical structures of evodiamine A-ring (**8**–**16**), E-ring (**17**–**28**), A, E-ring disubstituted (**29**–**36**), and D-ring derivatives (**37**–**40**).

**Scheme 2:**
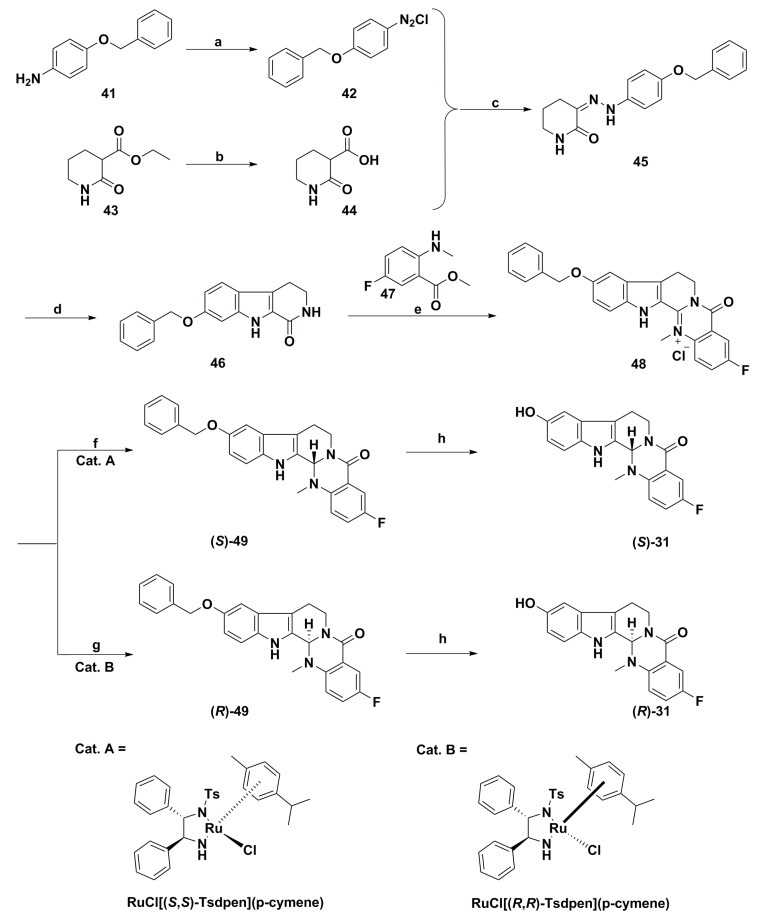
Synthetic route of (*R*)- and (*S*)-**31**. Reagents and conditions: (**a**) NaNO_2_, hydrochloric acid, 0–5 °C, 2 h; (**b**) KOH, rt, 12 h; (c) rt, 10 h; (**d**) HCOOH, reflux, 0.5 h; (**e**) POCl_3_, reflux, 3 days; (**f**) cat. A, HCOOH:Et_3_N (5:2), 0 °C, 8 h; (**g**) cat. B, HCOOH:Et_3_N (5:2), 0 °C, 8 h; (**h**) H_2_, 10% Pd/C, rt, 12 h.

Two years later, Dong et al. [169] synthesized a library of evodiamine A-ring (**8**–**16**), E-ring (**17**–**28**), A, E-ring disubstituted (**29**–**36**), and D-ring derivatives (**37**–**40**) (Figure 5). Taking the asymmetric center C-13b into account, they investigated the effects of C-13b chirality on the antiproliferative activity. Both *R*- and *S*-isomers of a highly active derivative **31** were synthesized (Scheme 2). In vitro results indicated compounds **8**, **11**, **18**, **19** and **31** exerted substantially increased cytostatic activity against HCT-116, MDA-MB-435 and A549 cell lines, with GI_50_ values lower than 3 nM. The C13b chirality showed distinct effects toward HCT-116 and A549 cells, (*S*)-**31** was slightly more active. Furthermore, these derivatives could induce apoptosis in A549 cells. Together with further computational calculations, these evodiamine derivatives could be used as dual inhibitors of topo I and topo II.

The mechanism of Topo I and Topo II inhibition suggested that test compounds (**11**, **33**, **35**, and **36**, Figure 5) acted as Topo II catalytic inhibitors. Moreover, at the dose of 1 mg/kg or 2 mg/kg, compounds **11** and **36** showed good antitumor efficacy and low toxicity in vivo. The SARs could be concluded as follows. Firstly, the 1-, 3-, 10- and 12-positions were favorable to be substituted, chlorine was more suitable than fluorine for both 3- and 10-positions. Free hydroxyl group was important for strong antiproliferative potency. Secondly, for the D-ring, changing the carbonyl group to the thiocarbonyl group at C-5 and the transformation of methyl group to an oxygen atom at N-14 showed positive effects of antiproliferative activity. Thirdly, some compounds of C-3, C-10 disubstituted possess synergistic effects. Compounds **31**–**33** and **36** with hydrophilic groups showed good antiproliferative activity and water solubility.

**Scheme 3:**
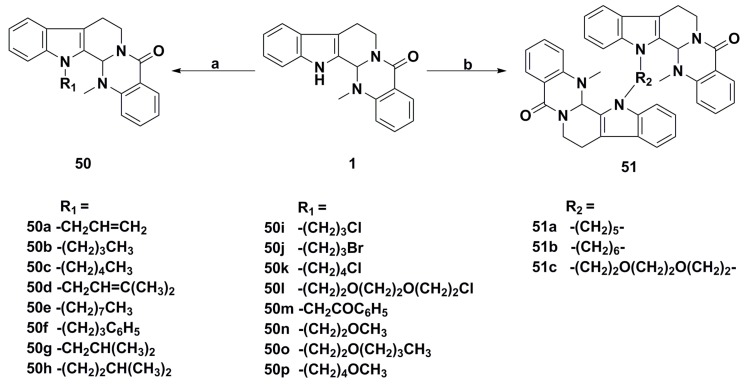
Synthetic route of evodiamine derivatives **50a**–**p** and **51a**–**c**. Reagents and conditions: (**a**) NaH, R_1_X, 8 h; (**b**) NaH, Br-R_1_-Br, 8 h.

In 2013, Song’s group designed and synthesized a series of evodiamine derivatives at N-13 (Scheme 3) [170]. They tested the cytotoxic activities of all derivatives against prostate cancer DU-145, PC-3, lung cancer H460, colon cancer HCT-5, glioblastoma SF-268 and MCF-7 cells. The results showed that **50b**, **50c**, **50i**, **50p** and **51b** exhibited stronger antiproliferative potency and broader spectra. Among them, **50p** was the strongest with IC_50_ values below 2 µM. The SARs indicated that the appropriate N-13 substitution of evodiamine resulted in higher cytotoxicity. The alkyl N-13 substituted groups decreased antiproliferative potency, and the derivatives with alkylamino-alkyl groups showed improved water solubility, but only moderate antiproliferative potency. The dimers exhibited moderate to good activity, and **50m** with a benzoylmethyl group was inactive. The apoptosis related mechanisms of compounds **50c**, **50p** and **51b** were also disclosed. Compounds **50c** and **51b** drastically induced apoptosis in MCF-7 cells at the early stage, while **50p** and **51b** induced apoptosis in HCT-15 cells at the late stage. Moreover, **50p** and **51b** induced late apoptosis in NCl-H460 cells with moderate increasing to 40–50%.

**Scheme 4:**
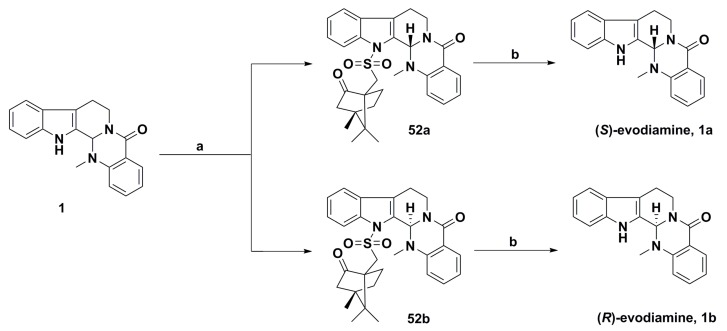
Synthetic route of evodiamine enantiomers **1a** and **1b**. Reagents and conditions: (**a**) (1*S*)-10-camphorsulfonyl chloride, NaH, 60 °C, 10 h; (**b**) NaOH, MeOH/THF 1:1, rt, 18 h.

**Scheme 5:**
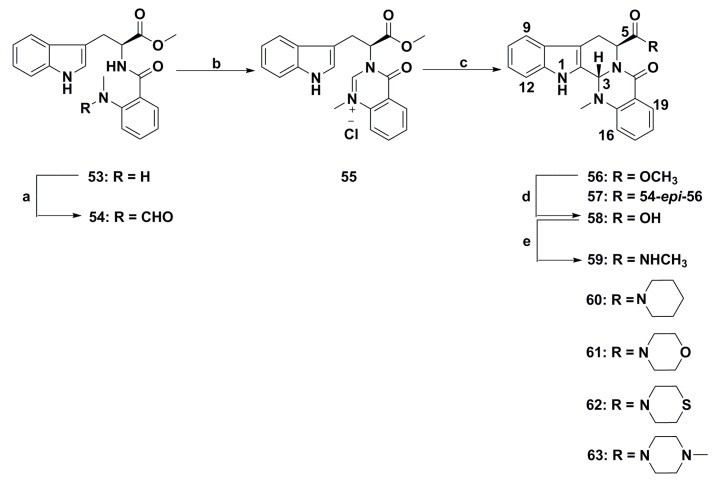
Synthetic route of C-ring modified (*S*)-evodiamine. Reagents and conditions: (**a**) HCOOH, (Ac)_2_O, rt; (**b**) HCl, dioxane, rt, 2 h; (**c**) pyridine, reflux, 2 h; (**d**) LiOH, reflux, 1.5 h; (**e**) various amines, HATU, DIPEA, rt.

In the same year, Nguyen’s group successfully isolated evodiamine enantiomers from 13 *Evodia rutaecarpa* samples by chiral high-performance liquid chromatographic method [171]. The results showed that *S*-(+)-evodiamine was present in higher concentration than *R*-(−)-evodiamine. Soon afterwards, Christodoulou et al. prepared the evodiamine enantiomers by chemical synthesis methods (Scheme 4), and synthesized a series of evodiamine C-ring modified derivatives (Scheme 5) [172]. In vitro antiproliferative assay showed that all compounds had low potency against H460, MCF-7 and HepG2 cells. However, (*S*)-evodiamine **1a** was the most active compound among them. Furthermore, in vitro Topo I inhibition of **1a** and **1b** showed effects only a high concentration of 500 μM. The human sirtuins SIRT1, SIRT2 and SIRT3 assays indicated *S* configuration was favorable.

Compounds **1a**, **1b**, **56**, ***ent*-56**, **58** and **59** showed better inhibitory effects on SIRT2 (60–80%) than SIRT1. In docking studies, they were well-fit to the binding pocket. Due to the steric hindrance, bulky substituents at position 5 had unfavorable poses, such as **60**, **61**, **62** and **63**. COOH, CONHCH_3_ and COOCH_3_ moieties at position 5 could increase the affinity towards sirtuins by additional hydrogen bonds. **1a**, **56** and ***ent*-56** also showed selectivity of SIRT2 over SIRT3. With this information in hand, evodiamine can act as a potential scaffold of sirtuins inhibitors.

**Figure 6:**
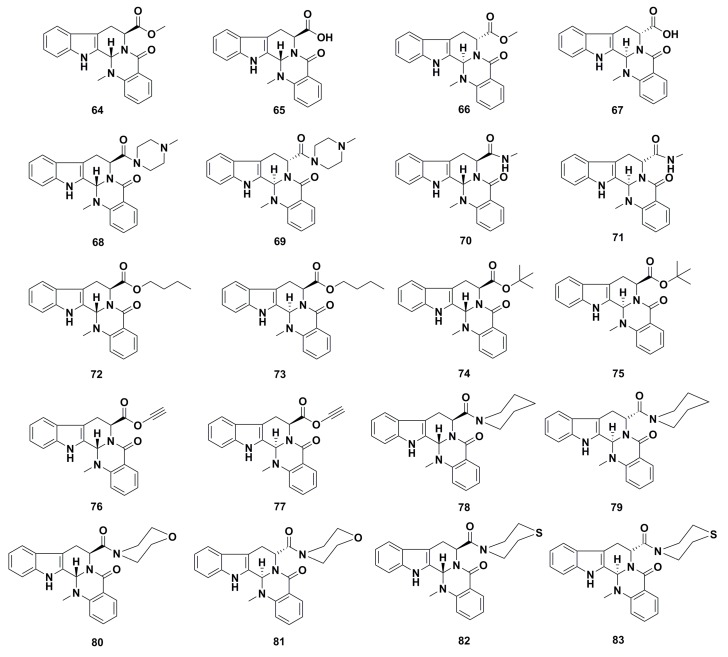
Chemical structures of synthetic evodiamine enantiomer analogues **64**–**83**.

In late 2013, a series of synthetic evodiamine enantiomer derivatives were synthesized by De Petrocellis et al. (Figure 6) [173]. The results showed the derivatives of *S*-(+)-evodiamine were more potent than *R*-(−)-evodiamine derivatives at human and rat transient receptor potential vanilloid type-1 (TRPV1) in transfected HEK-293 cells, with the corresponding EC_50_ values of 5 μM and 2 nM. Especially, a new lead analogue (**72**) was identified as the most potent TRPV1 agonist and better than the reference capsaicin. Previously, evodiamine was also reported as an agonist of TRPV1 in vitro and in vivo [174]. This work further confirmed evodiamine would qualify as potent TRPV1 agonists.

**Scheme 6:**
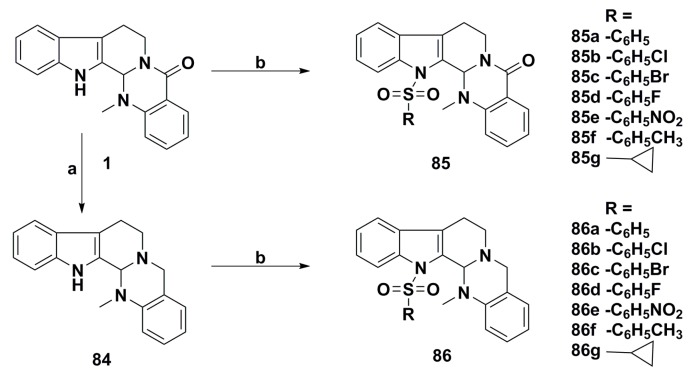
Synthetic route of evodiamine and 5-methylene evodiamine derivatives **85a**–**g** and **86a**–**g**. Reagents and conditions: (**a**) LiAlH_4_, rt, 8 h; (**b**) NaH, substituted benzenesulfonyl chloride or cyclopropanesulfonyl chloride, rt, 8–12 h.

In 2014, sulfonic acid esters and 5-methylene derivatives of evodiamine were designed and synthesized by Liang et al. (Scheme 6) [175]. **85e** exhibited more potent antiproliferative activity than evodiamine against A549, HepG2, U251, MCF-7 and HeLa cells, and **86a**–**g** showed higher IC_50_ values than the parallel compounds **85a**–**g**. The preliminary SARs suggested that *N*-*para* benzenesulfonylation was favorable for antiproliferative activity. The conversion from carbonyl group to methylene at C-5 would decrease the activities, and aromatic sulfonic acid esters were better than alkyl sulfonic acid esters for the antiproliferative activity. Moreover, when R was nitryl group, the activity decreased dramatically. In vivo antitumor assessment in the HepS xenograft showed **85e** could improve index of thymus and spleen and possessed less side-effect. Finally, according to in silico molecular docking, compounds **1** and **85a**–**g** showed high binding affinity with the target. Although **84** and **86a**–**g** had no binding affinity with the important residues of Topo I, they still exhibited antiproliferative activities. Between the ligand and the active site of Topo I, besides intermolecular H-bonds, other interactions might exist.

**Figure 7:**
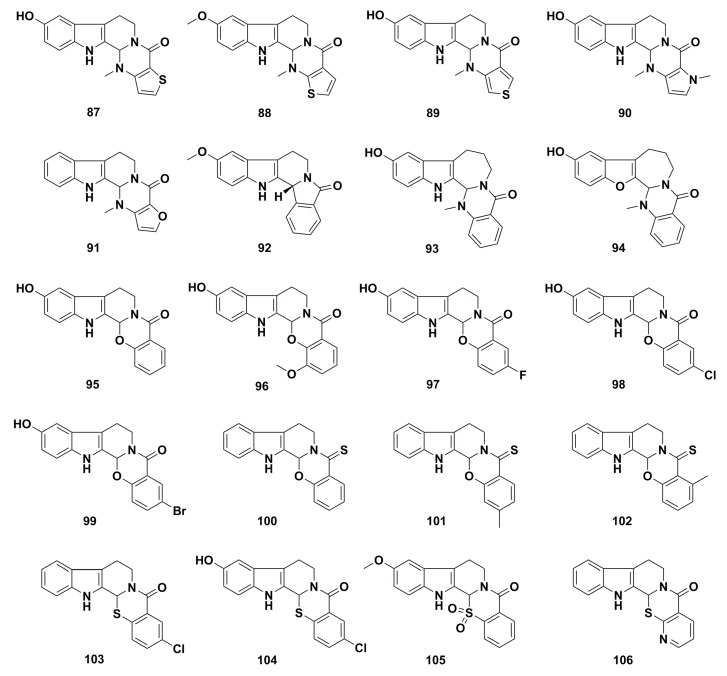
Chemical structures of evodiamine-inspired novel scaffold derivatives **87**–**106**.

In 2015, a library of 11 evodiamine-inspired scaffolds and their derivatives were obtained (Figure 7) [176]. Most of them showed antiproliferative activities against selected cancer cell lines. Among them, **104**, 3-chloro-10-hydroxyl thio-evodiamine, showed good antitumor efficacy and low toxicity. The mechanism and target studies indicated that **104** was the first-in-class Topo I/Topo II/tubulin triple inhibitor. The significance of this work could be concluded, that it provided the in-depth SAR of evodiamine inspired scaffolds, a series of potent antitumor molecules and the first-in-class Topo I/Topo II/tubulin inhibitors.

**Scheme 7:**
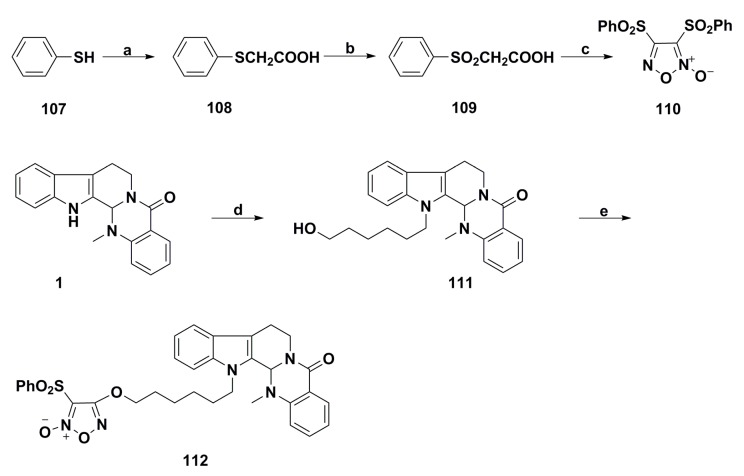
Synthetic route of nitric oxide donating evodiamine derivative **112**. Reagents and conditions: (**a**) ClCH_2_COOH, NaOH, 140 °C, 2 h; (**b**) H_2_O_2_, AcOH, rt, 3 h; (**c**) fuming HNO_3_, 90 °C, 4 h; (**d**) HO(CH_2_)_6_Br, NaH, rt, 3 h; (**e**) **110**, DBU, −15 °C, 3 h.

In 2016, Hua’s group published their first work in the field of the structural modification of evodiamine [177]. A series of nitric oxide donating evodiamine derivatives were designed and synthesized (Scheme 7). Of which, compound **112** showed good antiproliferative activity against A549, BGC-823 and human hepatoma Bel-7402 cells with IC_50_ values of 2.31, 0.07 and 2.10 µM, respectively. More importantly, **112** exhibited no cytotoxicity (>100 µM) against L-02 human normal liver cells. In Bel-7402 and BGC-823 cells, all the derivatives could release more than 75 µM/L NO at 1 h, and below 30 µM/L in L-02 cells. The mechanism study showed that **112** induced apoptosis and caused S phase cell-cycle arrest in Bel-7402 cells through caspase-dependent mitochondria related pathways. Western blot analysis revealed **112** could up-regulate proapoptotic Bax, FAS, cytochrome c and caspase-3, -8, -9, and down-regulate the Bcl-2 expression.

**Scheme 8:**
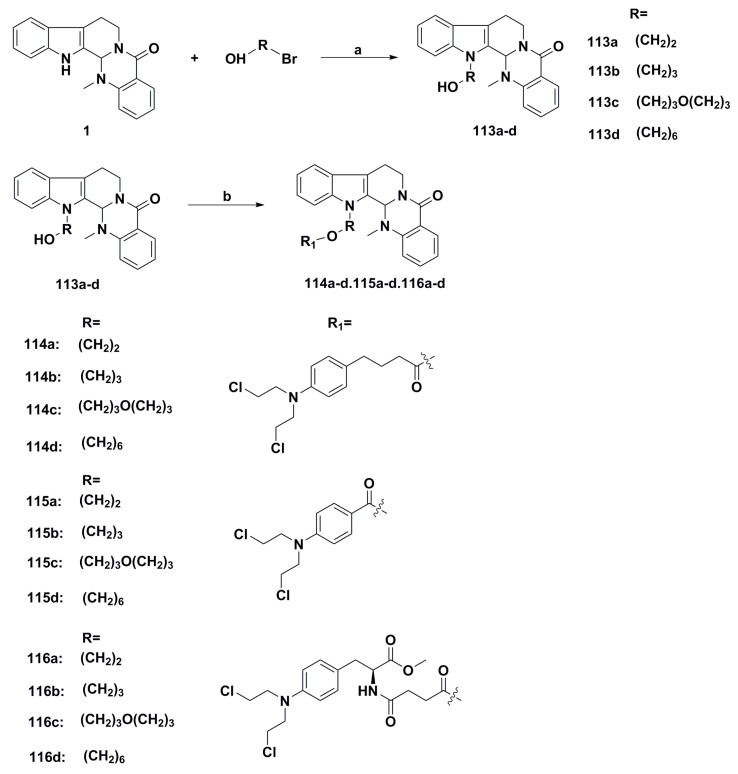
Synthetic route of nitrogen mustard derivatives (**114**–**116**) of evodiamine. Reagents and conditions: (**a**) NaH, rt, 3 h; (**b**) benzoic acid nitrogen mustard, methyl melphalan butyric acid or chlorambucil, EDCI, DMAP, rt, 12–24 h.

In 2017, this group continued structural modifications of evodiamine, and synthesized a series of nitrogen mustard derivatives of evodiamine (Scheme 8) [178]. The antiproliferative activities of **114a**–**d**, **115a**–**d** and **116a**–**d** against PC-3, HepG2, THP-1, HL-60 and PBMCs were tested. The results showed all the derivatives exhibited antiproliferative activities against tumor cells and no (>200 μM) antiproliferative activities against PBMCs. Among them, **116c** showed the strongest cytotoxicity against HL-60 and THP-1 cells with IC_50_ values of 0.50 and 4.05 μM, respectively. Further mechanism study manifested that **116c** could induce apoptosis and arrest cell cycle at G_2_/M phase via mitochondria-related pathways in HL-60 cells.

**Scheme 9:**
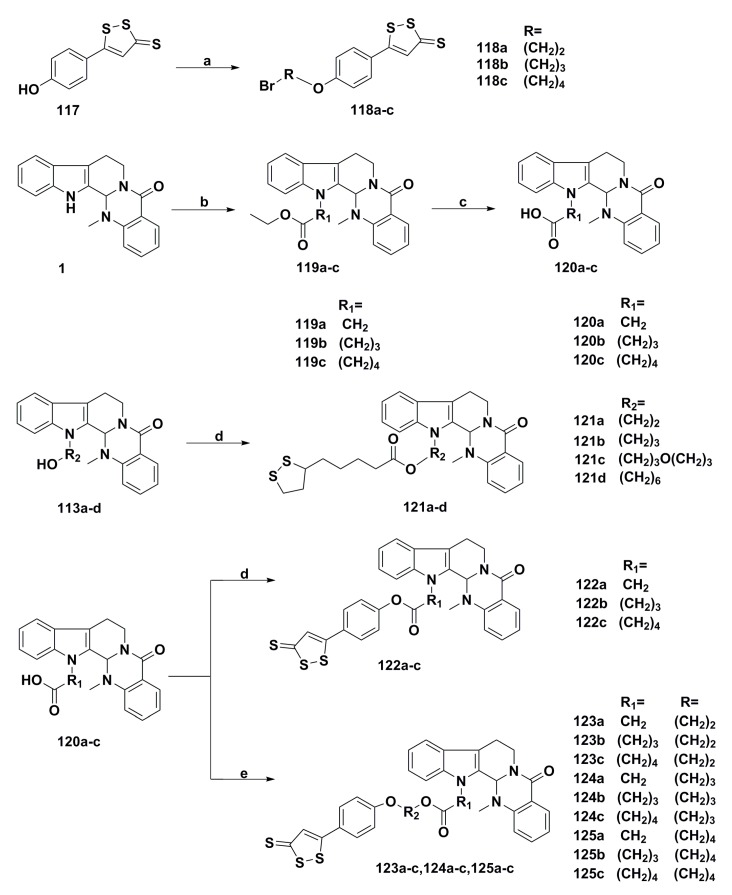
Synthetic route of H_2_S-donating derivatives (**121**–**125**) of evodiamine. Reagents and conditions: (**a**) Br(CH_2_)_n_Br, potassium carbonate, reflux, 8 h; (**b**) Br(CH_2_)_n_COOC_2_H_5_, NaH, 80 °C, 12 h; (**c**) potassium hydroxide, rt, 2 h; (**d**) *α*-thioctic acid, **117**, EDCI, DMAP, rt, 8–12 h; (**e**) **118a**–**c**, TEA, 80 °C, 8 h.

Recently, H_2_S donating derivatives of evodiamine (**121**–**125**) were further designed and synthesized (Scheme 9) [179]. Resulting hybrids were evaluated against Bel-7402, MCF-7, SGC-7901, human epithelial colorectal adenocarcinoma Caco-2 and HL-60 cells, and PBMCs for antiproliferative activities. Among these derivatives, compound **125c** showed the strongest potency against Caco-2 and HL-60 cells with IC_50_ values of 2.02 and 0.58 μM, respectively. Furthermore, compound **125c** possessed high selectivity between human normal PBMCs and HL-60 cells. The mechanism studies showed that **125c** could arrest HL-60 cell cycle at the G_2_/M phase and induce apoptosis. Western blot results confirmed the intrinsic apoptotic pathways.

## 4. Conclusions

The cytotoxic effects of evodiamine were correlated with its inhibitory activities against cell cycle progression, proliferation, invasion and angiogenesis, as well as apoptosis-induced effect. A variety of evodiamine derivatives have been synthesized to increase the antiproliferative effects of evodiamine. However, most of the studies were taken in in vitro and animal models, more clinical evidences regarding the effectiveness of evodiamine in the treatment of various types of human cancers are urgently needed. In addition, the low bioavailability limits the anti-cancer efficacy of evodiamine, novel evodiamine derivatives with enhanced bioavailability are necessary to be designed and synthesized. Further, development of new evodiamine delivery systems, such as a supermolecular nanoemulsion [180], or a phospholipid complex [40], might also be the appropriate strategies to increase the bioavailability of evodiamine in clinical practice.

## Abbreviations

AP-1	Activator protein 1
AIF	Apoptosis-inducing factor
AKT	Protein kinase B
aSMase	Acid sphingomyelinase
CSLCs	Cancer stem-like cells
DR	Death receptor
DNMT	DNA methyltransferase
EGFR	Epidermal growth factor receptor
EVO/HP-β-CD	Evodiamine into hydroxypropyl-β-cyclodextrin
ERK	Extracellular signal-regulated kinase
ER	Endoplasmic reticulum
ERCC1	Excision repair cross-complementing 1
Fas-L	Fas-ligand
HUVECs	Human umbilical vein endothelial cells
HCC	Hepatocellular carcinoma
HIF-1α	Hypoxia-inducible factor 1α
IL-1	Interleukin 1
JNK	c-Jun N-terminal kinase
JAK2	Janus kinase 2
LLC	Lewis lung carcinoma
L-OHP	Oxaliplatin
mTOR/S6K1	Mammalian target of rapamycin/p70 ribosomal S6 kinase 1
MAPK	Mitogen-activated protein kinase
miRNAs	microRNAs
MMP3	Matrix metalloproteinase 3
MDR	Multidrug resistance
Mcl-1	Myeloid cell leukemia 1
NF-κB	Nuclear factor-κB
NPs	Natural products
NSCLC	Non-small cell lung cancer
NO	Nitric oxide
NOTCH3	Neurogenic locus notch homolog protein 3
PARP	Poly (ADP-ribose) polymerase
PBMC	Peripheral blood mononuclear cell
PERK	Double-stranded RNA-activated protein kinase-like ER kinase
PTK	Protein tyrosine kinase
PLK1	Polo-like kinase 1
PKC	Protein kinase C
PPAR-γ	Peroxisome proliferator-activated receptor γ
ROS	Reactive oxygen species
SCLC	Small cell lung cancer
SHH/GLI1	Sonic hedgehog/GLI family zinc finger 1
STAT3	Signal transducer and activator of transcription signaling 3
SHP-1	Shatterproof 1
SARs	Structure-activity relationships
SIRT1	Silent information regulator of transcription
Topo	Topoisomerase
TRAIL	Tumor necrosis factor-α-related apoptosis‑inducing ligand
TGF-β1	Transforming growth factor-β1
TS	Thymidylate synthase
TRPV1	Transient receptor potential vanilloid type-1
VEGF	Vascular endothelial growth factor
WWOX	WWdomain-containing oxidoreductase
z-VAD-fmk	Benzyloxycarbonyl-Val-Ala-Aspfluoromethyl ketone
